# Cancers biliaires: aspects épidémiologiques cliniques et thérapeutiques à propos de 20 cas

**DOI:** 10.11604/pamj.2018.29.13.9922

**Published:** 2018-01-04

**Authors:** Ibrahima Ka, Magatte Faye, Papa Saloum Diop, Amadou Bocar Niang Aliou Coly Faye, Jean Marc Ndoye, Babacar Fall

**Affiliations:** 1 Service de Chirurgie Générale, Hôpital Général de Grand-Yoff, Sénégal

**Keywords:** Cancers voies biliaires, adécarcinomes, cholangiocarcinomes, morbidité, mortalité, Biliary tract cancers, adecarcinomas, cholangiocarcinomas, morbidity, mortality

## Abstract

Les cancers des voies biliaires se répartissent en deux localisations principales: les cancers de la vésicule biliaire qui sont des adénocarcinomes et les cholangiocarcinomes de localisations intra et extra-hépatiques. Il s'agit étude rétrospective portant sur 20 cas de cancers biliaires entre Janvier 2006 et Octobre 2014 à la clinique chirurgicale de l'Hôpital Général de Grand- Yoff. Il s'agissait de 40% de cancer de la vésicule biliaire, 60% de cancer de la voie biliaire principale. Le sex-ratio était de 1. L'âge moyen de nos patients était de 58,1 ans. Le délai diagnostique moyen était de 3,77 mois. La symptomatologie était dominée par le syndrome ictérique et les douleurs de l'hypochondre droit. Tous les patients avaient présenté un syndrome de cholestase biologique. L'échographie abdominale a été réalisée dans 65% des cas, la TDM abdominale dans 85% des cas et L'IRM dans 35% des cas. Les formes avancées étaient prédominantes dans notre série (n=19).La majorité de nos patients a bénéficié d'une chirurgie palliative. Le geste le plus pratiqué était la dérivation biliaire chez 50% des patients. On notait une prédominance des cholangiocarcinomes. La morbidité opératoire globale était de 43,75%. La mortalité globale toutes localisations confondues était de 31,25%. La moyenne de survie est de 4 mois et demi. Les cancers des voies biliaires sont d'expression multiforme, se différenciant essentiellement en un cholangiocarcinome intra, extrahépatique et adénocarcinome de la vésicule biliaire dont l'évolution est globalement différente mais le pronostic spontanément mauvais.

## Abstract (en)

Biliary tract cancers mainly occur in two sites: gallbladder cancer which are adenocarcinomas and intra- and extrahepatic cholangiocarcinomas. We conducted a retrospective study of 20 cases with biliary tract cancer in the Department of Surgery at the General Hospital in Grand-Yoff between January 2006 and October 2014. 40% of patients had gallbladder cancer, 60% of patients had common bile duct cancer. Sex ratio was 1. The average age of patients was 58.1 years. The average time to diagnosis was 3.77 months. Symptomatology was dominated by icteric syndrome and right hypochondrium pain. All patients had biological manifestation of cholestatic syndrome. Abdominal ultrasound was performed in 65% of patients, while abdominal CT scan in 85% of cases and MRI in 35% of cases. Advanced cancers were predominant in our case series (n=19). The majority of patients underwent palliative surgery. The most practiced treatment was biliary diversion (50% of patients). There was a predominance of cholangiocarcinomas. The overall operative morbidity rate was 43.75%. The overall mortality rate in our patients with biliary tract cancers of any site was 31.25%. Median survival was 4 months and a half. Biliary tract cancers have multifaceted features and can be differentiated essentially among intrahepatic cholangiocarcinoma, extrahepatic cholangiocarcinoma, gallbladder adenocarcinoma whose evolution is globally different but the prognosis is spontaneously poor.

## Introduction

Les cancers des voies biliaires se répartissent en deux localisations principales: les cancers de la vésicule biliaire qui sont des adénocarcinomes et les cholangiocarcinomes de localisations intra et extra-hépatiques. Ces derniers ont été particulièrement peu étudiés car il est difficile d'obtenir une preuve histologique de la maladie. Les données de registres sont donc particulièrement utiles pour étudier ces cancers rares. Leur incidence a été estimée à 2000 nouveaux cas par an en France, en 2000, soit environ 3% des cancers digestifs [1]. Ces taux, variables dans le monde selon les régions géographiques, ont augmenté au cours des 30 dernières années dans les pays occidentaux. Ils touchent, dans environ deux tiers des cas, les patients de plus de 65 ans. Le caractère sombre du pronostic est dû en partie à la découverte souvent très tardive de cette tumeur. La plupart des patients ont en effet une tumeur non résécable au moment du diagnostic. Actuellement la survie est inférieure à 5% à 5 ans. 90% des cholangiocarcinomes sont des adénocarcinomes. 60 à 70% sont des tumeurs à la bifurcation des canaux biliaires ou tumeurs de Klatskin, 20 à 30% des tumeurs de la voie biliaire principale, 5 à 10% des cholangiocarcinomes sont périphériques, provenant des petits canaux biliaires et se développant dans le parenchyme hépatique lui-même [2-5].

## Méthodes

Il s'agit d'une étude rétrospective allant du 01 Janvier 2006 au 31 Octobre 2014 portant sur 20 patients pris en charge dans le service de chirurgie générale de l'hôpital général de Grand-Yoff. Les données de l'étude étaient fournies par les registres du service d'hospitalisation. Les critères d'inclusion étaient: tout patient porteur d'un cancer des voies biliaires, disponibilité d'une observation médicale complète comprenant l'état civil du patient, son origine géographique, les signes cliniques, les différents examens complémentaires pratiqués, la thérapeutique instituée, l'évolution. Cependant les dossiers incomplets ou non retrouvés ont été exclus.

## Résultats

Dans notre étude, 40% présentaient un cancer de la vésicule biliaire (n=8), 60% un cancer de la voie biliaire principale (n=12). L'âge moyen était de 58,1 ans avec des extrêmes de 35 et 86 ans, un pic de fréquence globale qui se situait entre 50 et 60 ans. Pour les cancers de la vésicule biliaire le pic de fréquence se situait entre 50 et 60 ans et pour les cancers des voies biliaires principales deux pics de fréquence ont été observés entre 40 et 50 puis entre 60 et 70ans figure 2.La répartition selon le sexe était équitable avec 50% d'hommes et 50% de femmes et un sexe ratio égal à 1. Parmi les patients porteurs d'une tumeur de la vésicule biliaire, 75% étaient des femmes (n= 6), 25% des hommes (n=2) avec un sexe ratio de 0,33. Pour les tumeurs de la voie biliaire principale 66,67% des patients étaient des hommes (n=8), 33,33% des femmes (n=4) avec un sexe ratio de 2. Le délai diagnostique variait entre 0,5 mois et 9 mois avec un délai moyen de 3,77mois. La majorité de nos patients avaient consulté avant 6 mois. Les détails sont exposés au Tableau 1. La symptomatologie était dominée par le syndrome ictérique et les douleurs de l'hypochondre droit. Les données de l'examen physique étaient dominées par l'ictère cutanéo muqueux et l'hépatomégalie. Tous les patients avaient présenté une cholestase biologique, 60% avaient présenté une cytolyse. L'échographie abdominale était réalisée chez 65% de nos patients (n=13). La tomodensitométrie abdominale étaitréalisée chez 85% (n=17).L'imagerie par résonnance magnétique a été réalisée chez 35% de nos patients (n=7). Sur le plan histologique on notait une prédominance des cholangiocarcinomes. Dans le cadre du bilan d'extension, les formes avancées étaient prédominantes. La chirurgie à visée palliative était principalement effectuée. Aucun traitement complémentaire n'a été administré. La dérivation biliaire était effectuée chez 50% des patients (n=8); une segmentectomie V avec cholécystectomie était réalisé chez 6,25% de nos patients. La durée moyenne d'hospitalisation était 20,37 jours. La morbidité opératoire globale était 43,75 % (n=7). La mortalité globale était de 31,25% (n=5). La moyenne de survie est de 4 mois et demi.

**Tableau 1:** Répartition des patients en fonction du délai diagnostique

Délai diagnostique	Nombre de patients	Pourcentage
**Inférieur à 6 mois**	14	70
**Entre 6 et 9 mois**	5	25
**Supérieur ou égal à 9 mois**	1	5
Total	20	100

## Discussion

Le cholangiocarcinome représente 3% de l'ensemble des cancers digestifs. Il est la deuxième tumeur primitive du foie derrière le carcinome hépato cellulaire [6]. Le pic d'âge des patients avec cette maladie est la soixante-dixième année et il y a une légère prépondérance des hommes. Aux Etats-Unis, l'incidence rapportée est de 1 à 2 cas par 100 000 soit 3 500 nouveaux cas par an. L'élément nouveau de ces dernières années est l'augmentation significative de l'incidence du cholangiocarcinome intra-hépatique. Cette augmentation semble majeure en Grande-Bretagne, aux Etats-Unis, en Australie. Un peu moins significative en France, en Italie, en Japon. Il n'y a pas d'explication actuelle à l'augmentation très importante de l'incidence des nouveaux cas de cholangiocarcinomes intra-hépatiques. Cette augmentation ne semble pas retrouvée pour le cholangiocarcinome extra-hépatique dont l'incidence semble stable ou en très légère diminution [7-10]. Contrairement à ce qui se dit dans la littérature, ces cancers sont en très nette progression en Afrique noire. Ceci pourrait s'expliquer par le changement de mode de vie des populations et l'amélioration de l'accessibilité aux moyens diagnostics. L'âge moyen de nos patient toute localisations confondues était de 58,1 ans .Les cancers de la vésicule biliaire survenaient surtout entre 50 et 60ans, ceux de la voie biliaire principale entre 40 et 50 ans, puis entre 60 et 70 ans.

Dans la littérature Africaine, 50% des personnes atteintes de cancers de la vésicule biliaire en Algérie avaient moins de 55ans [11]. Ces cancers étaient cinq fois plus fréquents chez la femme que chez l'homme [11]. Dans notre série les patients sont plus jeunes, ceci pourrait s'expliquer par la jeunesse de la population en Afrique Noire. Nous nous attendions à un nombre plus important de cancers de la vésicule biliaire chez notre population féminine du fait de l'obésité et de son corollaire qui est la lithiase vésiculaire. Le nombre de cancers de la vésicule biliaire serait sous-estimé du fait des réalités culturelles et socio-économiques combinées à la limitation des moyens diagnostiques et thérapeutiques. La rectocolite hémorragique et la cholangite sclérosante primitive (CSP) représentent les facteurs de risque essentiels des cancers de la voie biliaire principale dans le monde occidental [12, 13]. Les autresfacteurs décrits sont : la lithiase biliaire intrahépatique (10% de risque), [14] les malformations biliaires congénitales comme la maladie de Caroli ou le kyste du cholédoque (15% de risque) les infections parasitaires et virales du foie [15-17]. La lithiase vésiculaire est le principal facteur de risque des CVB. Elle est retrouvée dans 70 à 98% des cas [18,19].

Dans notre série, comme dans celle de Peycru [20] aucun facteur de risque n'a été retrouvé Le délai diagnostic moyen était de 3,77 mois, néanmoins la majorité de nos patients avaient consulté avant 6 mois. Le délai diagnostic relativement long est dû au caractère insidieux de la maladie. En réalité les patients consultent après l'installation de l'ictère qui correspond en fait à un stade avancé de la maladie. La symptomatologie est dominée par l'ictère cutané-muqueux, et les douleurs de l'hypochondre droitune hépatomégalie. Comme par ailleurs dans la littérature [21], Tous nos patients présentaient une cholestase biologique dont 60% associée à une cytolyse. L'Alpha Foeto-Protéine était dosé chez 5 de nos patients (25%), il était élevé chez un seul d'entre eux. Le CA 19-9 était dosé chez 2 patients (10%), il était élevé chez un seul patient. L'échographie est un moyen d'imagerie non invasif, simple, reproductible, mais reste manipulateur dépendant. La sensibilité de l'échographie dans la détection d'une dilatation des voies biliaires et d'une obstruction des voies biliaires varie entre 55 et 91% [22]. L'échographie reste ainsi l'examen de première intention dans les cancers de la vésicule biliaire et suffit parfois à poser le diagnostic et à faire une première approche de l'extension de la lésion [23,24] La tomodensitométrie est une technique d'imagerie médicale moderne. Dans les cancers de la voie biliaire principale, la TDM est demandée en deuxième intention en complément de l'échographie en cas de non disponibilité de l'IRM. Dans les cancers de la voie biliaire principale, la TDM en plus de poser le diagnostic, permet de faire un bilan d'opérabilité.

Elle est plus performante dans la recherche de métastase à distance [24-26]. Dans notre étude elle a été réalisée chez 85% des malades (n=17) et a mis en évidence une dilatation des voies biliaires intra hépatiques dans 8 cas, une masse de la convergence hilaire chez 7 patients, des localisations secondaires hépatiques dans 6 cas, une grosse vésicule biliaire dans 5 cas, et un cas de vésicule scléro-atrophique. La bili-IRM ou cholangiographie par résonnance magnétique est le meilleur examen pour obtenir de façon non invasive une cartographie complète des voies biliaires intra et extra hépatiques. Il est très couteux et peu accessible. Dans les cancers de la voie biliaire principale elle permet de localiser avec précision l'obstacle (sensibilité supérieure à 95%) [27], de visualiser les structures canalaires exclues du fait d'une sténose, précisant ainsi l'extension locale et peut souvent identifier la nature de cet obstacle [28,29]. Dans notre étude, elle a été réalisée chez 35% de nos patients (n=7). Elle a mis en évidence une masse hilaire dans 5 cas, une dilatation des voies biliaires intra hépatiques dans 3 cas, 2 cas de métastases hépatiques, un cas de néoplasie de la vésicule biliaire et de sténose du cholédoque. Le bilan d'extension dépend de la localisation de la tumeur qui peut être vésiculaire, extra-hépatique, ou intra-hépatique. Le bilan d'extension locorégional des cancers de la vésicule biliaire est basé sur le scanner. L'IRM est indispensable au bilan d'extension des tumeurs extra et peri-hépatiques et a pour but d'en établir la résécabilité en évaluant le niveau de l'obstruction de la voie biliaire, l'envahissement intra-hépatique et l'extension aux structures vasculaires adjacentes [30]. Les extensions ganglionnaire et péritonéale restent les plus difficiles à évaluer quelle que soit la technique utilisée [31].

Dans notre série, la TDM abdominale avait mis en évidence chez trois patients des localisations secondaires hépatiques, l'IRM quant à elle avait montré ces métastases chez deux patients. La confirmation histologique n'est obtenue qu'après intervention chirurgicale. Dans notre étude, 16 patients ont bénéficié d'une exploration chirurgicale. Parmi ces patients explorés, 7 ont eu une biopsie et 4 une résection (cholécystectomie, segmentectomie). Sept résultats nous sont parvenus. On notait une prédominance des cholangiocarcinomes (n=7) corroborée par les données de la littérature [32]. Cependant dans une série Marocaine [33] les adénocarcinomes ont constitué 88,2% de la totalité des CVB diagnostiqués sur une durée de 5ans. Dans notre série 3 patients ont bénéficié d'une cœlioscopie exploratrice, 4 ont bénéficié d'une laparotomie exploratrice, deux d'entre eux ont bénéficié d'une segmentectomie V emportant la vésicule biliaire. Aucun de nos patients n'avait bénéficié d'un traitement adjuvant ou néo adjuvant. La morbidité opératoire globale était 37,5% (n=6). Dans la littérature les mêmes chiffres ont été pratiquement retrouvés, 40% dans la série d'E. Santoro et al [34], 43% dans la série de G. Ramacciato et al [35]. La mortalité opératoire était de 31,25% (n=5). Ramacciato et De Santoro trouvent respectivement 0 et 6,6%. La survie moyenne de 5 mois est nettement inférieure à celle des séries occidentales et pourrait s'expliquer par le mauvais état nutritionnel de nos patients, le retard diagnostic et l'absence de moyens thérapeutiques adéquats.

## Conclusion

Les cancers des voies biliaires sont d'expression multiforme, se différenciant essentiellement en cholangiocarcinome intra, extrahépatique et adénocarcinome de la vésicule biliaire dont l'évolution est globalement différente mais le pronostic spontanément mauvais. Une détection plus précoce, une meilleure compréhension des mécanismes de carcinogénèse, l'apport de nouvelles molécules anti-cancéreuses, ciblées sur ce type de tumeur, doit permettre d'améliorer significativement le pronostic dans les années futures.

### Etat des connaissances actuelle sur le sujet

- Les cancers des voies biliaires se répartissent en deux localisations principales: les cancers de la vésicule biliaire qui sont des adénocarcinomes et les cholangiocarcinomes de localisations intra et extra-hépatiques;
- Les cancers biliaires sont fréquents en occident et touche surtout les patients de plus de 65 ans;
- Leur pronostic reste sombre malgré les progrès sur le plan diagnostique et thérapeutique.

### Contribution de notre étude à la connaissance

- Les cancers biliaires sont une réalité en Afrique particulièrement au Sénégal et sont l'apanage du sujet jeune;
- Le délai diagnostique très long explique les formes avancées et le pronostic péjoratif;
- Dans notre pratique, en l'absence d'écho endoscopie et de biopsie systématique, le scanner occupe une place centrale dans la stratégie diagnostique.

## Conflits d’intérêts

Les auteurs ne déclarent aucun conflit d'intérêts.
